# Food and Drug Analysis

**DOI:** 10.3390/molecules25102403

**Published:** 2020-05-21

**Authors:** Ping-Chung Kuo

**Affiliations:** School of Pharmacy, College of Medicine, National Cheng Kung University, Tainan 701, Taiwan; z10502016@email.ncku.edu.tw; Tel.: +886-6-2353535 (ext. 6806)

Food can be regarded as functional if it beneficially affects one or more target functions in the body in a way that is relevant to either the state of well-being and health or to the reduction of the risk of a disease [1]. It is also indicated that a functional food is any food that may provide a health benefit beyond the traditional nutrients it contains. An important concern of these functional foods is to provide an appropriate dose of bioactive components in order to have a beneficial rather than a toxic effect on human health [2]. Since the market and consumers have more and more interests in the health-enhancing role of specific foods and physiologically active food components, functional foods have received renewed attentions [3]. Functional foods are usually developed with specific health effects, including but not limited to anti-aging, anti-inflammation, and cardiovascular protection. Therefore, this Special Issue, “Food and drug analysis”, wishes to include the various aspects of exploring natural sources as healthy food and characterizing the molecular structures of bioactive principles.

In this issue, Wu et al. [4] reported one review that covered the anti-inflammatory and anticancer properties of bioactive compounds from *Sesamum indicum* L. and provided a common scope of discovery and development of lead compounds from natural sources. The use of foodstuff as natural medicines has already been established through studies demonstrating the pharmacological activities that they exhibit. *Sesamum indicum* L. is composed of lignans such as sesamin, sesamol, sesaminol and sesamolin, and these lignans have been widely studied and are known to possess antiaging, anticancer, antidiabetes, anti-inflammatory and antioxidant properties. Modern chronic diseases, which can transform into clinical diseases, are potential targets of these lignans. The prime example of chronic diseases is rheumatic inflammatory diseases, which affect the support structures and the organs of the body and can also develop into malignancies.

Other articles related to *Callicarpa hypoleucophylla*, *Briareum excavatum*, *Allium mongolicum*, and *Taiwanofungus camphoratus* were also good examples of studies on natural food sources for the possible new drug candidates. Plants of the genus *Callicarpa* are known to possess several medicinal effects. Two new clerodane-type diterpenoids along with seven known compounds were isolated from the leaves and twigs of the Taiwan endemic plant *C. hypoleucophylla* and then characterized. The anti-inflammatory activity of isolated compounds based on the suppression of superoxide anion generation and elastase release was evaluated. Among the isolates, some compounds showed anti-inflammatory activity by suppressing superoxide anion generation and elastase release [5].

Five 8,17-epoxybriaranes, including three new compounds along with two known analogues, were isolated from the octocoral *Briareum excavatum*. Their structures were elucidated by spectroscopic methods, including 1D and 2D nuclear magnetic resonance (NMR) studies and high resolution electrospray ionization mass spectrometry (HRESIMS) analysis. One briarane-type isolate exerted inhibition effects on inducible nitric oxide synthase (*i*NOS) and cyclooxygenase-2 (COX-2) release from RAW 264.7 [6].

Dong et al. [7] designed to isolate and identify the flavonoids and phenols from the aerial parts of *Allium mongolicum* Regel by using various chromatographic and spectrophotometric methods, a bioassay on motility of mouse isolated intestine tissue, as well as qualitative analysis using liquid chromatography/mass spectrometry (LC–MS). The aim of this study is to clarify whether these isolates have the effect of improving gastrointestinal function. As a result, six new flavonoid glycosides and four new phenolic acid glycosides along with twenty-one known compounds were characterized. Among them, eleven flavonoids and three phenolic acids showed significant increase in the height of mouse small intestinal muscle. Furthermore, according to the retention time and the exact mass-to-charge ratio (*m*/*z*), thirty-one compounds were unambiguously identified by comparing to the standard references by using LC–MS. According to these results, a fast analysis method for flavonoids and phenolic acids in *A. mongolicum* was established.

Hung et al. [8] reported some idea against the tumor resistance from natural food sources. Resistance to anti-cancer drugs is one of the main factors of treatment failure resulting in high morbidity. Among the reasons of resistance, the overexpression of efflux pumps leading to multidrug resistance is an important issue that needs to be solved. *Taiwanofungus camphoratus* has been used as a nutritional supplement to treat various cancers. In addition to the four new constituents reported, the major isolates zhankuic acids A–C were evaluated for their P-glycoprotein (P-gp) inhibitory effects and the results showed that zhankuic acid A was the most potent. This study provides support for the use of *T. camphoratus* in the further development of cancer therapy.

Curcumin is the naturally occurring phytochemical from the rhizome of *Curcuma longa* L. It is a polyphenol with a symmetrical structure composed of two ortho-methoxyphenol rings connected to each other through a flexible conjugated hydrocarbon chain. Lee et al. [9] reported a series of derivatives modified from the curcumin di-*O*-2,2-bis(hydroxymethyl)propionate that shows significant in vitro and in vivo inhibitory activity against MDA-MB-231 cells with eight to ten-fold higher potency than curcumin. The established structure–activity relationship and pharmacokinetic outcomes are the guidance for future development of 4,4-disubstituted curcuminoid 2,2-bis(hydroxymethyl)-propionate derivatives as anticancer drug candidates.

Molecular hydrogen (H_2_) has been shown to have antioxidant and anti-inflammatory activities that may reduce the development and progression of many diseases. In the study reported by Yao et al. [10], hydrogen-rich water (HRW) was obtained by reacting hybrid magnesium–carbon hydrogen storage materials with water. Then, the effects of intake of the HRW on the activities of xenobiotic-metabolizing enzymes, membrane transporters, and oxidative stress in rats were investigated. The results from this study suggest that the consumption of HRW may not affect xenobiotic metabolism or oxidative stress in liver. However, the intake of HRW may increase the efflux of xenobiotics or toxic substances from the liver into bile by enhancing *p*-glycoprotein and Mrp2 protein expressions.

The liquid chromatography methods including ultrahigh performance liquid chromatography (UHPLC) or coupled with mass spectrometry (LC/MS) provided feasible methods for the analysis of the natural healthy foods or potential medicinal plants. Identification and quantification of polyphenols in plant material are of great interest since they make a significant contribution to its total bioactivity. High-resolution mass spectrometry (HRMS), which is able to provide the accurate mass of unknown compounds, has become an important tool for characterizing chemical components in natural products. In the study reported by Chiriac et al. [11], a UPLC-Orbitrap-MS/MS approach using the variable data acquisition mode was developed and applied for separation, identification, and quantification of the main polyphenolic compounds in *Medicago sativa* L. and *Trifolium pratense* L. sprouts in different germination stages.

Tölgyesi et al. [12] described a liquid chromatography tandem mass spectrometric (LC–MS/MS) method for analyzing five *Alternaria* toxins in sunflower oil. An optimal sample preparation condition was achieved when samples were dissolved in *n*-hexane and extracted with methanol/water mixture, followed by sample pre-concentration with solvent evaporation. This study is focusing only on this lipophilic matrix and in using all corresponding isotopically labeled internal standards (ISTD) to compensate the matrix effect that strongly influences the LC–MS/MS analysis of toxins.

Exposure to residues of antibiotics and insecticides in aquacultured food can adversely affect humans and animals and thus affect public health globally. Hence, Chang et al. [13] used a validated LC–MS/MS and gas chromatography tandem mass spectrometric (GC–MS/MS) method to examine the levels of residues of 12 sulfonamides as well as 18 organophosphorus insecticides in aquacultured fish in Taiwan. According to the experimental results, the risk of exposure to sulfonamide and organophosphorus insecticide residue by consuming aquacultured fish in Taiwan was thus negligible, signifying no immediate health risk related to the consumption of fish. Residue levels in fish must be continually monitored to further determine the possible effects of these residues on human health.

Pan et al. [14] established the geographical origin traceability in *G. straminea* by analyzing its chemical profiles assisted with a UPLC-Q exactive mass spectrometer, from which 43 compounds were identified by comparing retention times and mass spectrometry. A total of 42 samples from different habitats was determined by a UPLC-Q exactive mass spectrometer and the data were assayed with multivariate statistical analysis. Eight characteristic compounds were identified to determine the geographical origin of the herb. To estimate the key characteristic markers associated with pharmacological function, the inhibiting activities of nitric oxide (NO) production in lipopolysaccharide (LPS)-induced macrophages were also examined. These findings are crucial in the determination of botanical origin and evaluation of the quality of *G. straminea*.

Lee at al. [15] developed an in vitro tyrosinase inhibition assay in combination with ultraperformance liquid chromatography-orbitrap mass spectrometry (UPLC-orbitrap-MS) for the rapid screening and identification of tyrosinase modulators from roots of *Angelica keiskei*. The present study indicated that the combination of in vitro tyrosinase inhibition assay coupled with UPLC–MS/MS could be widely applied to the rapid screening of active substances from various natural resources.

*Lepidium meyenii* is now widely consumed as a functional food and medicinal product, which is known as an enhancer of reproductive health. However, the specific chemical composition and mechanism of action for improving sexual function are unclear. Gao et al. [16] aimed at screening and determining the potential compounds, which promote mouse leydig cells (TM3) proliferation. The results suggested that three compounds had good activities on the proliferation of TM3 and promoting testosterone secretion, which might be the potential bioactive markers related to the enhancing sexual ability functions of *L. meyenii*. This study also provided the reference for a simple, quick method to screen the promoting Leydig cell proliferation active components in traditional Chinese medicine (TCM).

In addition to the extensively applied chromatographic methods, nuclear magnetic resonance (NMR) spectroscopy is also used in screening for novel bioactive molecules. All these new analytical methods accelerate the research and make the potential targets available in the near future. Hachem et al. [17] reported an easy, rapid and accurate ^1^H-NMR analytical method to establish the chemical profiles of red yeast rice dietary supplements and to quantify their monacolin contents. The total content of monacolin was close to that measured by UHPLC, as shown by the good linear correlation between the two sets of values.

The rapid dispersion of new psychoactive substances (NPS) presents challenges to customs’ services and analytical laboratories, which are involved in their detection and characterization. When the seized material is limited in quantity or of a complex nature, or when the target substance is present in very small amounts, the need to use advanced analytical techniques, efficient workflows and chemo-informatics tools is essential for the complete identification and elucidation of these substances. Tsochatzis et al. [18] described the application of such a workflow in the analysis of a single blotter paper, seized by Swedish customs, that led to the identification of a lysergic acid diethylamide (LSD) derivative, 1-butyl-lysergic acid diethylamide (1B-LSD). Its identification was made possible by comprehensively combining gas chromatography with mass spectrometry detection (GC–MS), liquid chromatography coupled with high-resolution tandem MS (LC–HR-MS/MS), Orbitrap-MS and both 1D and 2D nuclear-magnetic-resonance (NMR) spectroscopy. All the obtained data have been managed, assessed, processed and evaluated using a chemo-informatics platform to produce the effective chemical and structural identification of 1B-LSD in the seized material.

Colombo et al. [19] discussed the advances in the analysis of veterinary drug residues in food matrices by capillary electrophoresis (CE) techniques offered a sensitive and fast analytical technique and guided a new way to the safety concern related to the natural foods. Over the years, the availability of different modes, interfaces, and formats has improved the versatility, sensitivity, and speed of CE techniques. Thus, CE represents a powerful tool for the analysis of a large variety of food matrices and food-related molecules with important applications in food quality and safety. This review focuses the attention of CE applications over the last decade on the detection of different classes of drugs with a potential risk for animal and human health. In addition, considering that the different sample preparation procedures have strongly contributed to CE sensitivity and versatility, the most advanced sample pre-concentration techniques are discussed here.

This issue wishes to provide an intellectual platform for scientists to publish their results covering the topics of bioactive constituents, biological activities, and analytical methodologies in relation to food, drugs, and herbal medicines. The above-mentioned articles form a solid base for future discussions regarding the development of natural healthy foods and the improvement of currently employed analytical methods. Finally, the guest editor wishes to express sincere gratitude to all the authors for their impactful contribution as well as all the reviewers who assisted to ensure high scientific quality of the content.
